# Lessons from Polyomavirus Immunofluorescence Staining of Urinary Decoy Cells

**DOI:** 10.3390/life13071526

**Published:** 2023-07-07

**Authors:** Sahra Pajenda, Zsofia Hevesi, Michael Eder, Daniela Gerges, Monika Aiad, Oliver Koldyka, Wolfgang Winnicki, Ludwig Wagner, Farsad Eskandary, Alice Schmidt

**Affiliations:** 1 Division of Nephrology and Dialysis, Department of Internal Medicine III, Medical University of Vienna, 1090 Vienna, Austria; sahra.pajenda@meduniwien.ac.at (S.P.); michael.eder@meduniwien.ac.at (M.E.); monika.aiad@students.boku.ac.at (M.A.); farsad.eskandary@meduniwien.ac.at (F.E.); alice.schmidt@meduniwien.ac.at (A.S.); 2 Center for Brain Research, Medical University of Vienna, 1090 Vienna, Austria; szofia.hevesi@meduniwien.ac.at; 3 Division of Endocrinology and Metabolism, Department of Internal Medicine III, Medical University of Vienna, 1090 Vienna, Austria; oliver.koldyka@meduniwien.ac.at

**Keywords:** BK-polyomavirus, decoy cell, polyoma nephropathy, large T antigen, VP1

## Abstract

Decoy cells that can be detected in the urine sediment of immunosuppressed patients are often caused by the uncontrolled replication of polyomaviruses, such as BK-Virus (BKV) and John Cunningham (JC)-Virus (JCV), within the upper urinary tract. Due to the wide availability of highly sensitive BKV and JCV PCR, the diagnostic utility of screening for decoy cells in urine as an indicator of polyomavirus-associated nephropathy (PyVAN) has been questioned by some institutions. We hypothesize that specific staining of different infection time-dependent BKV-specific antigens in urine sediment could allow cell-specific mapping of antigen expression during decoy cell development. Urine sediment cells from six kidney transplant recipients (five males, one female) were stained for the presence of the early BKV gene transcript lTag and the major viral capsid protein VP1 using monospecific antibodies, monoclonal antibodies and confocal microscopy. For this purpose, cyto-preparations were prepared and the BK polyoma genotype was determined by sequencing the PCR-amplified coding region of the VP1 protein. lTag staining began at specific sites in the nucleus and spread across the nucleus in a cobweb-like pattern as the size of the nucleus increased. It spread into the cytosol as soon as the nuclear membrane was fragmented or dissolved, as in apoptosis or in the metaphase of the cell cycle. In comparison, we observed that VP1 staining started in the nuclear region and accumulated at the nuclear edge in 6–32% of VP1^+^ cells. The staining traveled through the cytosol of the proximal tubule cell and reached high intensities at the cytosol before spreading to the surrounding area in the form of exosome-like particles. The spreading virus-containing particles adhered to surrounding cells, including erythrocytes. VP1-positive proximal tubule cells contain apoptotic bodies, with 68–94% of them losing parts of their DNA and exhibiting membrane damage, appearing as “ghost cells” but still VP1^+^. Specific polyoma staining of urine sediment cells can help determine and enumerate exfoliation of BKV-positive cells based on VP1 staining, which exceeds single-face decoy staining in terms of accuracy. Furthermore, our staining approaches might serve as an early readout in primary diagnostics and for the evaluation of treatment responses in the setting of reduced immunosuppression.

## 1. Introduction

Among kidney transplant recipients under immunosuppressive therapy, uncontrolled reactivation or primary infection with the human polyomaviruses 1 (BK-Virus, BKV) and less often 2 (John Cunningham, JC-Virus, JCV) can turn into a common complication that is mostly observed within the early phase after transplantation [1]. Patients may develop increasing viremia that can result—if not timely reversed by lowering immunosuppression—into functional decline of the kidney transplant and ultimately lead to polyomavirus-associated nephropathy (PyVAN), with a substantial degree of advanced early graft loss [2]. The routine screening for BKV/JCV via PCR in blood is currently thought to represent the gold standard and has become a mainstay of post-transplant surveillance in most centers [3]. To date, the primary response to a sustained increase in viral replication is the concerted reduction of immunosuppression that, in the majority of cases, will lead to resolving viremia. In refractory cases, as some small interventional studies have shown, the administration of foscarnet, cidofovir, leflunomide and/or intravenous immunoglobulins (IVIG) can be used, although the overall level of evidence for these approaches is quite low [4]. Experimental approaches such as adaptive virus-specific T-cell therapy exist; however, no other therapeutic solutions are yet known in routine clinical practice [5].

Polyomavirus transmission occurs via the fecal-oral route in childhood, but normally healthy individuals show no symptoms during BKV/JCV primary infection. Overall, 83 to 90% of adults in Europe show a robust humoral immune response against these polyomaviruses as a sign of prior exposition to this pathogen [6]. However, in a low percentage of healthy individuals (0–6%), intermittent viruria without viremia can be observed, which has not been shown to be associated with any pathologic process [7,8]. On the contrary, immunocompromised patients are more likely to show symptomatic infection and viremia. Thereby, kidney transplant recipients tend to develop BK-viremia and less often JC-viremia, although rare cases of JCPyVAN have been described, which are thought to follow a more latent disease course compared to BKPyVAN [7,9]. In those patients, BK/JC-viremia typically follows an antecedental constant rise of viruria. The test for polyomavirus copy number in plasma therefore has a high sensitivity for identifying patients at risk of developing PyVAN [10,11,12].

BK virus-transformed decoy cells appear in urine, which can be identified via cytology and quantified as a percentage of total urine cells [4,13]. These so-called decoy cells in urine can serve as a specific surrogate for BKV/JC viremia and PyVAN, but show an overall low sensitivity in panoptic staining, which increases moderately with the amount of decoy cells in urine. However, infected cells identified by double immunostaining allow a more specific diagnosis of BKPyVAN [14]. In this case, decoy cells are former renal epithelial cells that have been infected and have undergone a process that has allowed BKV/JCV to replicate, and thus the cell moves from the resting phase to the S phase of the cell cycle. This is achieved by inhibiting the retinoblastoma tumor suppressor via binding to the large tumor antigen (lTag) and the associated effects on host-cell DNA and virus genome replication [15,16,17] by using the host’s replisome [18]. As a result, the nucleus develops a disproportionate nuclear/cytoplasmic ratio during the transition from the G_0_ to the S phase of the cell cycle, which has also been incorporated into the current pathologic hallmarks of cytopathic changes often seen in kidney transplant biopsies with PyVAN [19,20,21]. Further morphological changes are nuclear inclusions and shiny staining [22]. The major site of BK replication is the proximal tubule epithelial cell, but as shown by in vitro experiments, BK infection can cause marked changes in gene transcript and protein expression by two days after infection [23]. This affects proteins involved in energy metabolism, detoxification, tumor necrosis factor signaling and protein translation. However, it has also been shown that infection can occur in uroepithelial cells further distal from the renal tubular cells, such as the urinary bladder [14,22].

The double-stranded 5000 bp genome of the BKV or JCV encodes early transcribed genes, such as the lTag, and late-transcribed genes, of which VP1 is the major capsid protein that forms the outer layer of the viral capsid [24,25]. The lTag protein is translated in the nucleus shortly after infection of the cell and forms hexameric structures that bind to the viral genome ORI with their origin-binding domain [26,27], initiating replication of the BK- or JC viral genome. Transcription of the VP1 gene is initiated later, and virus particle formation may be initiated soon thereafter.

We hypothesized that staining various time-dependent polyomavirus-specific proteins in the urine sediment of patients with known viremia and assessing their localization in cellular compartments during different phases of the cell cycle could enable us to identify a tool to assist clinicians in making treatment decisions, such as reduction of immunosuppression or as yet unknown antiviral treatments. In this pilot project, we focused on lTag as the most important member of the early transcriptional part of the BKV genome, and especially on the VP1 protein as the most important part of the late transcriptional part. VP1 staining was used to follow virus particle synthesis and its passage through the cell and its environment. We evaluated the expression and distribution pattern of lTag and VP1 by immunostaining and confocal microscopy in urine sediment cells of patients with PyVAN.

## 2. Patients and Methods

### 2.1. Patients

Five male and one female kidney transplant patients aged 51–61 years, diagnosed with BK viremia, had undergone allograft biopsy because of an acute increase of sCr combined with BK viremia and urinary decoy cells.

Morning urine sediment was obtained and separated into two portions. One part was used for cytopreparations as described below. The other part was lysed in TRIzol and frozen at −20 °C for further analysis.

### 2.2. Virus Genotype Definition

The total RNA was isolated by phase separation by adding 200 µL chloroform to the TRIzol lysate and spinning at 12,000× *g* for 10 min. The aqueous phase was taken off and mixed with 650 µL of isopropanol, out of which the RNA was precipitated by pelleting at 12,000× *g* for 10 min. Following a wash with 75% ethanol, the semidry RNA pellet was redissolved in nuclease-free water and used for cDNA transcription. In brief, 800 ng of total RNA was mixed with random primers in a volume of 12 µL and heated for 3 min at 65 °C, followed by chilling in ice water. The dNTP, reaction buffer, Ribolock and RNA Aid were then added and 5 min annealing at 25 °C was followed by cDNA synthesis at 42 °C for 60 min. The process was stopped by heating the sample at 80 °C for 10 min.

As primers for the VP1 PCR reaction, the forward primer ATGGCCCCAACCAAAAG and reverse primer TTAAAGCATTTTGGTTGCAATTG were used. The cycling conditions were initially 5 min denaturing at 94 °C followed by 30 cycles at 94 °C for 30 s denaturing, 55 °C for annealing and 68 °C for 60 s for synthesis. The resultant PCR product was then submitted for Sanger sequencing.

### 2.3. Cytopreparations

Morning urine was collected in a sterile container and aliquots of 7 mL were centrifuged at 2000× *g* for 10 min. The supernatant was discarded and the cell pellet was resuspended in 1 mL of culture medium (RPMI supplemented with 10% calf serum). Next, 70 µL of the resulting cell suspension was added to the funnel of the cytocentrifuge and spun at 1200 rpm for 3 min. The cytopreparation was air-dried and subsequently fixed in acetone for 5 min. The slide was then wrapped in aluminum and frozen at −25 °C. For immunostaining, the frozen slides were thawed under air flow, maintaining the aluminum wrapping until ambient temperature was reached to prevent water condensation and protein denaturation.

### 2.4. Immunofluorescence

After thawing the cytoslides, a hydrophobic circle was drawn around the cell-containing area and 30 µL of PBS was applied to this area to rehydrate the preparation. After rehydration, approximately 60 µL of the antibody solution was applied to the cytopreparation, which was incubated overnight in a humidified chamber at 4 °C on a shaking platform. For detecting the large T-antigen, the affinity purified anti-SV40 large T-antigen-specific antibody (diluted 1:100) PAS 112036 was used (Invitrogen, Waltham, MA, USA), and for staining BK polyoma virus VP1, the monoclonal (diluted 1:30) MAB:33242 clone 4942 (Invitrogen) was used. The next morning, the cytopreparation was washed in PBS with constant agitation of the wash liquid. Then, the secondary antibody solution Alexa-Fluor 488 goat anti rabbit IgG (A11008) or Alexa-Fluor 594 goat anti mouse IgG (H+L) (A11032, Invitrogen) was applied to the labeled areas containing the cells. After 60 min incubation in the moist chamber, the slides were washed in PBS for 10 min with constant stirring. For nuclear DNA staining, a three-minute staining with DAPI was performed before washing. Finally, an embedding solution and a glass coverslip were applied before images were recorded using an Axiovert confocal microscope.

## 3. Results

We used reverse transcription of RNA from the urine sediment of six BKPyVAN patients (Table 1), and VP1 transcripts from the late coding region of BK polyomavirus were amplified by PCR and sequenced. Thereby, BK virus genotype 1 was identified in all six patients. Confocal microscopy of these urine sediment cells was used to localize the product of the early coding region lTag and the late coding portion VP1. For this purpose, cytopreparations were stained by immunofluorescence.

### 3.1. LTag Distribution

**Figure 1:**
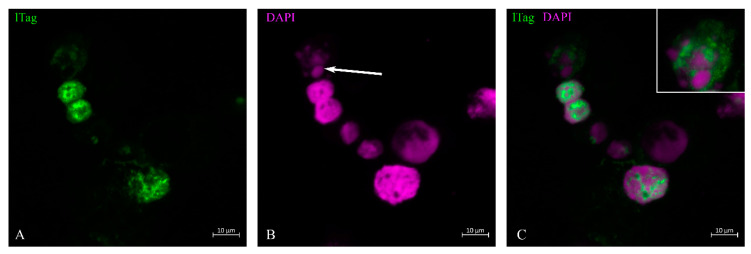
Cytopreparation of urine sediment and lTag immunofluorescence staining. Out of eight tubular epithelial cells, six presented morphological disparities: three presented with disproportionately enlarged nuclei, two with moderately enlarged nuclei and one with apoptotic bodies. Two cells with moderately enlarged nuclei and the decoy cell in the lower part of the figure showed distinct lTag nuclear staining (**A**). In the upper part of the image, the cell shows apoptotic bodies (long-tailed arrow, (**B**)). This part of the image is shown as an insert at higher magnification in (**C**). In this cell, the lTag staining has spread from the apoptotic bodies into the cytosol. Cells with normal-sized nuclei are weakly lTag-positive at specific sites of the nucleus (**C**).

**Figure 2:**
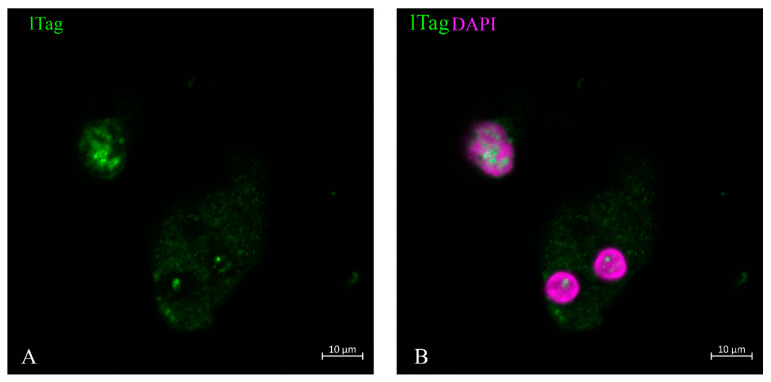
Ltag-positive tubular cells at different stages of the cell cycle. The cell in late telophase, or representing an endocycling cell with absence of cell division but with already separated nuclei, still had cytosolic staining and incipient nuclear spot staining at a specific location (**A**). The cell in the upper part showed nuclear spiderweb-like staining, but its nuclear chromatin did not show a clear border and shaded out into the cytosol (**B**).

**Figure 3:**
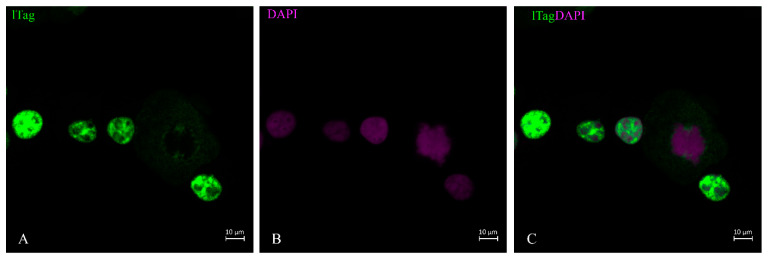
Green monkey kidney cell line ltag staining. Cos cells, known to be positive for the SV40 lTag, showed the typical strict nuclear staining pattern in variable intensity, but this staining spread into the cytosol (**A**,**C**) when the nuclear membrane was dissolved, such as at the metaphase stage of the cell cycle shown by DAPI stain (**B**).

LTag staining was performed on urine sediment when about 20% of urine sediment cells resembled decoy cells. The distribution of lTag took place within the nucleus (Figure 1). It began at specific locations within the nucleus when the nucleus still maintained its normal size (Figure 1 and Figure 2). The distribution spread over the nucleus in a ‘spiderweb’ fashion (Figure 1). During the phase when nuclear staining was most intense, little staining was found in the cytoplasm (Figure 2 and Figure 3). When the nuclear membrane dissolved in the mitotic cycle (Figure 2 and Figure 3A,C) or was destroyed in apoptosis (Figure 1C, insert), lTag staining distributed into the cytoplasm. This redistribution of the protein was typically observed in the green monkey kidney cell line COS. The nuclear lTag in its variable staining intensity was distributed in a reticular pattern, but was seen in the cytoplasm when the metaphase chromosome plate was formed (Figure 3).

### 3.2. VP1 Translation and Distribution

**Figure 4:**
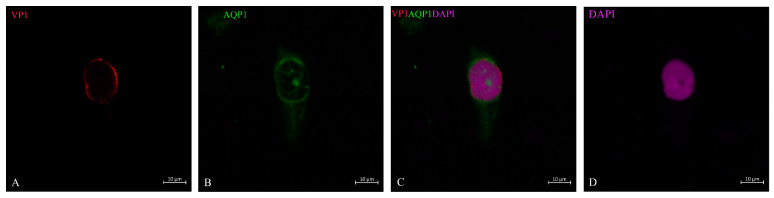
Encapsulation and production of the virus in the nucleus based on VP1 staining. Proximal tubule epithelial cell showed weak, homogeneous VP1 staining throughout the nuclear region, but with higher intensity at the nuclear margin (**A**); the cell was further defined by AQP1 staining (**B**). It is not clear whether the virus had passed through the nuclear pores or was concentrated in the outer region of the nucleus (**C**). This represented an early stage of infection, which was representative for 16–20% of VP1-positive cells in these patients. This is a representative picture, which was observed in such a status of virus replication from these patients. DAPI staining is shown in (**D**).

**Figure 5:**
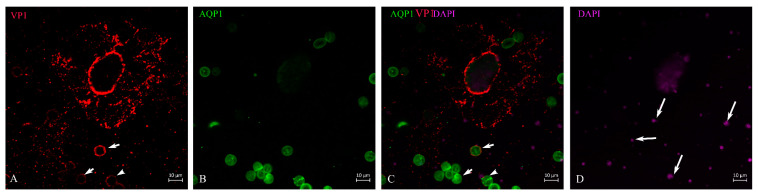
BK virus distribution within the cell and surroundings. Proximal tubular epithelial cell with AQP1 staining (**B**) over the nucleus. VP1 staining at the outer edge of the nucleus was retained, whose cytosol might have partly disintegrated and VP1-positive virus particles were distributed in a web-like fashion in exosome size inside of the cell and the area around it (**A**). This nucleus exhibited reduced DNA staining by DAPI (**D**). Erythrocytes known to be positive for AQP1 (**B**,**C**) were marked by VP1 (short-tailed arrows (**A**)) because of membrane binding of the BK virus. Apoptotic bodies floating around in the urine at the time of decoy cell collection (DAPI long-tailed arrows (**D**).

**Figure 6:**
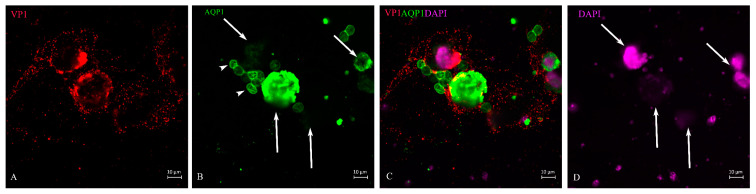
BK virus spreading in the surroundings of ghost cells. Partially disrupted proximal tubule epithelial cells and VP1-positive viral particles spreading around the cells in an exosome-like size (**A**). The ghost cells showed different intensities of AQP1 staining (green, long-tailed arrows (**B**,**C**)) and erythrocytes (short-tailed arrows (**B**)). DNA staining by DAPI (**D**) showed separated apoptotic bodies and the greatly reduced nuclear staining in the ghost cells (long-tailed arrows (**B**,**D**) and the fragmented nucleus in the upper part of the image (**D**).

**Figure 7:**
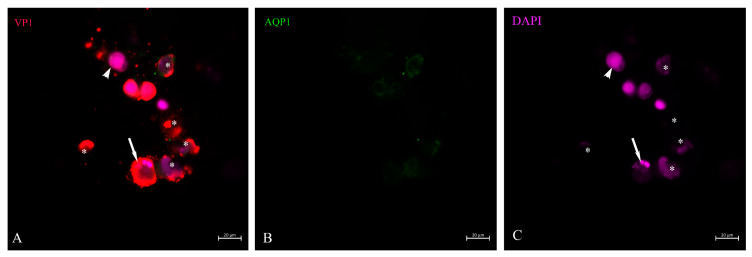
VP1 and AQP1 immunostaining of urine sediment decoy cells in asynchronous stage of infection. A cluster of VP1-positive staining cells (**A**). AQP1 as marker for proximal tubular cells (**B**). Small green particles represent cell fragments (**B**). One of the VP1-positive staining cells (**A**) showed apoptotic bodies indicated by a long-tailed arrow (**C**) and reduced DNA staining of the entire nuclear area. This is a cell in the late stage of infection. The cell with enlarged nucleus (short-tailed arrow (**C**)) in the upper part showed low VP1 stain (**A**) and might therefore be in the early phase of infection and virus replication (**A**). Six ghost cells with reduced nuclear DNA staining (**C**) still showed positive VP1 staining (**A**) and are marked with an asterisk (*).

After lTag transcription and translation was deployed, further steps represent the production of virus capsid proteins. The outer layer of the capsid is the VP1, which is supplemented by VP2 and VP3 in the inner compartment of the virion. Its transcription and translation is achieved by host-cell enzymes in a strictly concerted mode. As already demonstrated, in the case of viruses, this can happen within the nucleus of the host cell [28], or the proteins are shuttled back into the nucleus from the cytosol due to the nuclear localization signal. We were able to show that in decoy cells of human BKPyVAN patients, the VP1 staining in cells with intact morphology could be restricted to the nucleus and its outer rim where it is concentrated (Figure 4A,C). As shown in the cell (Figure 4), the outer rim of the nucleus underwent some sort of demarcation and it was not entirely clear whether the virus particles had already passed the nuclear membrane. About 16–20% of VP1-positive cells were found at this stage of virus infection depending on the patient and specimen (Table 2). In contrast to these cells were decoy cells with a disintegrated nucleus and reduced DNA staining by DAPI, such as in Figure 5, or in an apoptotic nucleus (Figure 6 and Figure 7), where VP1 staining was distributed all over the cytoplasm (Figure 5) and seemed to represent a late stage of infection. In general, these cells were characterized by much stronger VP1 staining and represented the dominant version of the positive cells (80–84%, see Table 2). The staining intensity and thereby virus particle content showed remarkably different extent and differed from cell to cell. On rare occasions, these cells even appeared in clusters (Figure 7) and were positive for AQP1 as a marker of proximal tubular origin. In most of them, the cytoplasmic membrane appeared to be destroyed or had lesions.

When the cytoplasmic membrane was ruptured, the virus particles dispersed in the environment, probably encapsulated in exosomes (Figure 6). When also present in urine, we found that some of these exosome-like particles attached to erythrocyte membranes (Figure 5, short-tail arrows) and stained the membrane edge to very different degrees. This is consistent with the fact that in earlier experiments, the erythrocyte agglutination test has been used in human polyomavirus research [29]. The VP1 staining of tubular epithelial cells was highly variable (Figure 7), indicating the asynchronous infection stage of these urinary sediment cells. Some of them could not be identified as decoy cells by simple H/E staining. The disproportionately enlarged nucleus did not go along with high VP1 expression (Figure 7). DAPI staining of the nuclear DNA was frequently reduced and nuclear borders were not clearly demarcated in up to 40% of decoy cells, and apoptotic bodies (arrow in Figure 7) were seen in up to 10% of decoy cells which stained for VP1. Fragments of cells were found all over the cyto-preparation which contained less than 10% VP1 staining (Figure 6).

### 3.3. VP1 and lTag-Positive Decoy Cells in Process of Apoptosis

Apoptosis, programmed cell death, is a specific process that can either be induced by certain types of immune cells, e.g., when the expression of danger signals or microbial/viral proteins on target cells is recognized by them (extrinsic), or under circumstances when distinct intracellular signals shift the overall cellular response towards the apoptosis pathways (intrinsic). In the case of polyomavirus-infected renal epithelial cells, they will often become targets of specific cytolytic T cells and NK cells upon recognition by an adequate immune response [30,31,32]. The number of late-stage apoptotic cells with highly condensed chromatin and nuclear DNA distributed in apoptotic bodies varies from day to day. However, it must be assumed that these cells received the hit while intact and subsequently exfoliated from the basement membrane and shed. Such cells are still positive for lTag, but apoptotic bodies are negative; rather, the protein is distributed throughout the cytosol (Figure 1C insert). As expected, these apoptotic cells were positive for VP1 to varying degrees (Figure 6 and Figure 7). In addition, apoptotic bodies floated in the urine (Figure 6), which entered the urine sediment through centrifugation conditions, as indicated in the Methods section, but these were negative for VP1 staining. Such a scenario was caused by VP1-positive cell ghosts that have spread their entire nucleus except for a minimal DNA residue, and from which VP1-positive small microsomes were released (Figure 6). Such mode of virus release was observed in all six patients, but the percentage of decoy cells with such virus-spreading conditions was not more than 3–4%.

## 4. Discussion

In this work, we focused on urinary sediment cell morphology and its relationship to early gene expression in terms of lTag, and the late gene product VP1 as an indicator for BK polyomavirus production and particle formation. This question was investigated by using lTag and virus capsid specific antibodies and observing the lTag protein distribution and the VP1 protein that forms the outer layer of the virion by confocal microscopy.

Firstly, we focused on the lTag as the early transcribed gene of the polyoma virus, incorporating a nuclear localization sequence similar to that of the SV40 virus [33], and therefore detected within the nucleus starting shortly after nuclear separation in the telophase. It spread in a ‘spider web’ fashion all over the nucleus in resting cells at specific stages of infection. We could show that it was redistributed into the cytosol when the nuclear membrane was dissolved, such as in the cell cycle, or destroyed, as in apoptosis. Whether lTag staining in apoptotic or mitotic cells detected the intact lTag protein or only a fragmented form or a specific splice variant [34] must be left open for this method.

In a second step, regarding gene translation from the late coding region [24] and virion assembly, it was clearly demonstrated, confirming the work of previous authors [35], that virus production is initiated and accomplished mainly in the nucleus. Subsequently, the virus particles spread through the cytosol and were distributed in the environment in exosome-sized vesicles. Earlier work has associated the promyelocytic leukemia nuclear bodies with JCV production sites and have termed these “virus factories” [36]. This morphology of viral spread, as shown by this work, has not yet been demonstrated, but previous authors have suggested that the BK polyoma virus hides in extracellular vesicles [37], so-called exosomes, to evade immune defense. This could help explain why the virus can still spread even when patients are positive for BK antibodies. Embedding of JC polyomavirus in extracellular vesicles has been shown by earlier authors [38], and our morphologic work supports this theory.

The third important observation of this work is that erythrocyte membranes are susceptible to BK virus binding, which could serve as a vehicle for transport of virions, which then leads to randomly distributed foci of BKPyVAN in kidney transplant recipients. If this erythrocyte binding is also true for the JC virus, this could help explain the transmission of virions from the kidney to the brain.

Previous authors have used dual immunocytochemistry to identify the origin of decoy cells and whether they originate from the ureterorenal region or urinary bladder [14]. We used AQP1 as a marker for proximal tubular cells [39,40].

Testing urine sediment for decoy cells has, without debate, gained importance, and various methods, such as Sternberg–Malbin staining, have improved availability and show excellent discriminative potential from other cells of the urinary tract [41,42]. Combined confocal immunostaining for proteins specific to the early and late stages of infection will extend the specificity for counting polyomavirus-infected cells beyond simple decoy staining. Various stages of apoptotic cells and ghost cells can be detected as virus carriers. These would be overlooked in panoptic decoy cell staining. However, it must remain open whether the extremely high viral replication in them, where the virus completely takes over the transcription and translation machinery, has led to intrinsic apoptosis. Nevertheless, extrinsic apoptosis caused by cytolytic immune cells remains another option. In particular, this could occur after modification of immunosuppression.

According to our data, the combination of a high number of decoy cells, especially when identified by immunostaining with BK-virus-specific antibodies, in conjunction with the BK virus copy number in the plasma, seems to be a strong indication of an ongoing infection of the allograft with BK virus. This is true, especially when the copy number reaches 10,000 copies per mL [43]. It is not yet decided whether this can replace the allograft biopsy and serve as a liquid biopsy in the future. Because the BK virus infection sites in the allograft are unevenly and patchily distributed, BK immunostaining in fine-needle biopsies can miss the infection in up to 10–30% of cases [10].

Another important question is whether the timing of a biopsy and decision-making is significantly facilitated by these two non-invasive testing methods. This question cannot be answered clearly in this study. However, follow-up of patients with proven BKPyVAN undergoing modified immunosuppression shows a decreasing number of decoy cells in the urine and, with some delay, a decrease in viral copies in the plasma. Consequently, the two methods combined, plasma copy numbers and immunostained decoy cells in urine, should be able to assess the status of viral replication in the allograft. However, we noted decoy cells in low percentages (1–4%) for several months while allograft function was stable. The cells in which the BK virus replicates could also originate from a site distal to the nephron. But still, this is important as the virus exhibits oncogenic potential and BK-positive urothelial carcinomas are frequent among transplant patients [44], or an already ongoing BK-virus-induced malignant process might be the origin of exfoliation [31].

This work stresses that monitoring decoy cells by urine cytology and virus-specific immunostaining is important information for the care of kidney transplant patients. Routine hematoxylin/eosin examination of decoy cells misses some of the infected cells, especially when large amounts of inflammatory cells are present, which is observed in about 20% of kidney transplant patients for various reasons, e.g., concurrent asymptomatic urinary tract infections.

## 5. Limitations of This Work

Immunostaining with mAbs is complicated by the specificity of the antibodies, which may result in missing BK mutants with rare genotypes. In addition, the virus replication rate in the cells must reach a certain level for the staining intensity to reach the detection limit. Although the antibody used works excellently in patients positive for the BK polyomavirus genotype 1, it is not clear whether this will be the case in other genotypes.

## 6. Conclusions

Diagnostics need to become more specific, as much more has been learned in recent years about the twelve characterized members of the human polyomavirus and the specific association with human disease. Therefore, immunostaining of decoy cells in the urine sediment for routine diagnostics needs to be further explored and improved. This might become of significant value for clinical application.

## Figures and Tables

**Table 1:** Demographic data of the six kidney transplant patients. ADPKD: autosomal dominant polycystic kidney disease, BK PCR: BK polyomavirus PCR, eGFR: estimated glomerular filtration rate, KTX: kidney transplantation, NP: nephropathy, * 95% granulocytes. BK PCR data are given in copy numbers per ml blood plasma.

	Case 1	Case 2	Case 3	Case 4	Case 5	Case 6
Age	61	58	52	60	51	60
Gender	m	m	m	m	m	f
Underlying disease	ADPKD	ADPKD	Reflux NP	Diabetic NP.	Undefined	Undefined
eGFR (MDRD) mL/min	15	32	12	42	24.51	24.51
BK PCR (Plasma)	5.2 × 10^4^	4.1 × 10^5^	1 × 10^2^	2.3 × 10^4^	4 × 10^3^	3 × 10^8^
Decoy cells in urine (%)	50	20	30	40	90	5 *
Months after KTX	6	2	96	27	4	14

**Table 2:** Percentage of VP1 staining cells at different stage of virus production. Faint nuclear staining with intact morphology was much less frequent than cells with nuclear fragmentation and membrane damage resembling ghost cells.

	Start of Virus Production at Nucleus (%)	Ghost Cells Membrane Damage (%)
Case 1	6	94
Case 2	18	82
Case 3	20	80
Case 4	16	84
Case 5	21	79
Case 6	32	68

## Data Availability

All data ae provided within this publication.
